# Olive Tree (*Olea europeae* L.) Leaves: Importance and Advances in the Analysis of Phenolic Compounds

**DOI:** 10.3390/antiox4040682

**Published:** 2015-11-03

**Authors:** Leila Abaza, Amani Taamalli, Houda Nsir, Mokhtar Zarrouk

**Affiliations:** Laboratoire de Biotechnologie de l’Olivier, Centre de Biotechnologie de BorjCedria B.P. 901, 2050 Hammam-Lif, Tunisia; E-Mails: abaza1tn@yahoo.com (L.A.); houda.nsir@gmail.com (H.N.); zarrouk.mokhtar@gmail.com (M.Z.)

**Keywords:** olive leaves, phenolic compounds, advanced analytical techniques

## Abstract

Phenolic compounds are becoming increasingly popular because of their potential role in contributing to human health. Experimental evidence obtained from human and animal studies demonstrate that phenolic compounds from *Olea europaea* leaves have biological activities which may be important in the reduction in risk and severity of certain chronic diseases. Therefore, an accurate profiling of phenolics is a crucial issue. In this article, we present a review work on current treatment and analytical methods used to extract, identify, and/or quantify phenolic compounds in olive leaves.

## 1. Olive Leaves as a Potential Source of Phenolic Compounds

Phenolic compounds, ubiquitous in plants, have been shown to exhibit a wide range of physiological properties, such as anti-allergenic, anti-artherogenic, anti-inflammatory, anti-microbial, antioxidant, anti-thrombotic, anti-cancer, cardioprotective, and vasodilatory effects [1]. A possibility of obtaining these interesting components is their extraction from natural matrices and another interesting approach is the extraction of such compounds from the food industry byproducts, which are usually discarded or employed to produce animal feed [2].

Olive (*Olea europaea* L.) is one of the most important crops in the Mediterranean countries. More than eight million ha of olive trees are cultivated worldwide among which the Mediterranean basin presents around 98% of them [3]. *Olea europaea* L. is widely studied for its alimentary use, the fruits and the oil are important components in the daily diet of a large part of the world’s population [4]. Both the cultivation of olive trees and olive oil extraction generate every year substantial quantities of products generally known as “olive byproducts” and having no practical applications. Olive leaves, available throughout the year, are one of the byproducts of olive farming; they accumulate during the pruning of the olive trees (about 25 kg of byproducts (twigs and leaves) per tree annually) and can be found in large amounts in olive oil industries after being separated from fruits before processing (about 10% of the weight of olives) [2]. Several reports have shown that olive leaves have antioxidant activity [5,6,7,8,9,10], anti-HIV properties [11], anti-proliferative and apoptotic effects [12], protective effect against human leukemia [13], lipid-lowering activity [7], *etc.*

The leaf is the primary site of plant metabolism at the level of both primary and secondary plant products [14] and can be considered as a potential source of bioactive compounds [15]. Numerous studies have been focused on the composition of olive leaves based on phenolic compounds considering their richness of such valuable compounds.

Phenolic compounds in olive leaves are numerous and of diverse nature. They are grouped with regard to major molecular characteristics as simple phenols and acids, lignans, secoiridoids and flavonoids [15], including flavones (luteolin-7-glucoside, apigenin-7-glucoside, diosmetin-7-glucoside, luteolin, and diosmetin), flavonols (rutin), flavan-3-ols (catechin), substituted phenols (tyrosol, hydroxytyrosol, vanillin, vanillic acid, and caffeic acid), and oleuropein [16]. Oleuropein, related secoiridoids, and other derivatives are the principal compounds of olive leaves [17] among which the major compound frequently reported is oleuropein. Flavonoids may occur in appreciable amounts [18]. Simple phenols and acids are present in lower amounts. However, several factors may influence the qualitative and quantitative phenolic composition of olive leaves among which we can cite date of collection [19], drying conditions [20], cultivation zone [21], extraction procedure [21,22], and cultivar [22,23].

**Figure 1:**
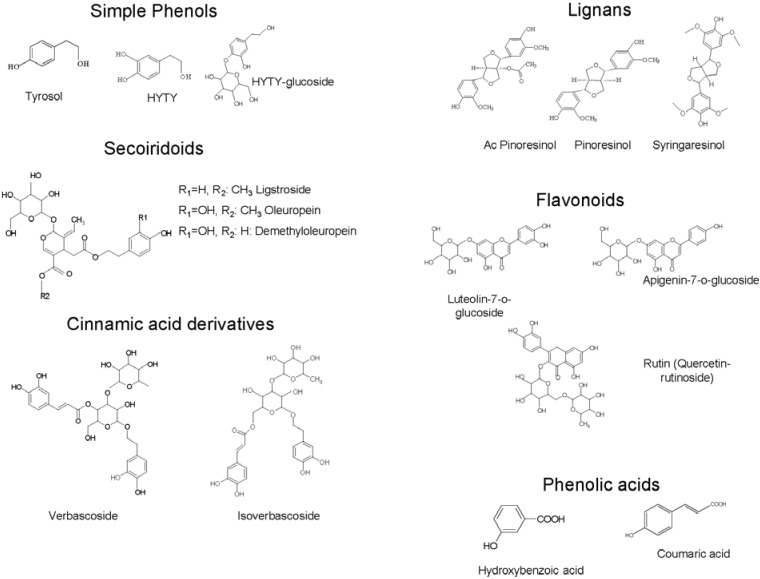
Phenolic classes and structures of main phenolic compounds in olive leaves.

In Figure 1 the main classes and structures of some phenolic compounds in olive leaves are presented.

## 2. Sample Preparation

After collection, fresh olive leaves are washed with distilled water to eliminate any traces of dust. In order to stabilize the byproduct and to avoid quality losses and undesirable degradation during storage and transportation, the immediate dehydration of olive leaves is the most important operation in post-harvest processing [24]. The leaves have to be dried for use as a food additive [25]. They are often dried before extraction of valuable compounds to reduce their moisture content and to avoid the interference of water on the process [26]. The drying process should be undertaken in closed and controlled equipment to improve the quality of the final product [26]. However drying is a notoriously energy-intensive operation that easily accounts for up to 15% of all industrial energy usage, often with relatively low thermal efficiency in the range of 25% to 50% [27].

Traditional methods of drying, such as shade or sun drying, are still practiced for drying. However this operation is not well controlled which may influence the final quality of the product. For industrial purposes, hot air drying is the most widely used method, since it allows an accurate control of the process variables [24]. Several researches have been interested in the investigation and modeling of the drying behavior of olive leaves. Modeling is an interesting tool to evaluate and quantify the effect of process variables on drying rate and useful information may be obtained about the mechanisms involved [27]. It is necessary for anticipating the drying time and product moisture content, developing new products, designing the appropriate equipment, and optimization of the process [28].

In Table 1, we give some drying processes reported in the literature for olive leaves.

**Table 1:** Drying process for olive leaf dehydration.

Objectives of the Research	Drying Process and Conditions	Reference
Study the effect of blanching and/or infrared drying on the color, total phenols content and the moisture removal rate of four olive leaf varieties	**Infrared dryer** Infrared drying temperatures: 40, 50, 60 and 70 °C	[29]
Investigate the main effects of process variables on the product quality during heat pump drying of olive leaves Determine an optimum process conditions for drying of olive leaves in a pilot scale heat pump conveyor dryer	**Pilot scale heat pump conveyor dryer** Drying air temperature range: 45–55 °C Drying air velocity range: 0.5–1.5 m/s Time range: 270–390 min	[30]
Investigate the main effects of process variables on the product quality during hot air drying of olive leaves Determine an optimum process conditions for drying of olive leaves in a tray drier	**Laboratory-type tray dryer** Drying compartment dimensions: 0.3 × 0.3 × 0.4 m Drying air temperatures: 40–60 °C Drying air velocities: 0.5–1.5 m/s Process time: 240–480 min	[31]
Study the influence of the ultrasound power application during the drying of olive leaves in the kinetics of process	**Pilot scale convective dryer modified to apply power ultrasound** Drying temperature: 40 °C Air velocity: 1 m/s Levels of electrical power applied to the ultrasound transducer: 0, 20, 40, 60 and 80 W Ultrasonic power density in drying chamber: 0, 8, 16, 25 and 33 kW/m^3^	[27]
Determine and test the most appropriate thin-layer drying model. Reveal the effects of drying air temperature and velocity on the effective diffusion coefficient and activation energy for understanding the drying behavior of olive leaves	**Thin-layer dryer** Drying air temperatures: 50, 60 or 70 °C Drying air velocities: 0.5, 1.0 or 1.5 m/s	[28]
Investigate the effect of solar drying conditions on the drying time and some quality parameters of olive leaves particularly the color, total phenol content and radical scavenging activity	**Laboratory convective Solar Dryer** Drying air temperatures: 40, 50 and 60 °C Drying air velocities: 1.6 and 3.3 m^3^/min	[26]
Develop a direct and rapid tool to discriminate five Tunisian cultivars according to their olive leaves by using FT-MIR spectroscopy associated to chemometric treatment	**Microwave** Two times for 2 min Maximum power 800 W (2450 MHz)	[32]
Study the effect of freezing and drying of olive leaves on the antioxidant potential of extracts Choose an appropriate drying process to obtain extracts rich in bioactive compounds	- **Hot air drying by forced air laboratory dryer** 70 °C for 50 min and at 120 °C for 12 min Air flow: 0.094 m^3^/s Air velocity: 0.683 m/s -**Freeze air drying by freeze dryer chamber** Initial temperature: −48 ± 2 °C, shelf temperature set at 22 ± 2 °C. Time: 48 h and Pressure: 1.4 × 10^−1^ mbar	[33]

It was reported that the infrared drying induces a considerable moisture removal from the fresh leaves (more than 85%) and short drying time (varying from ≈162 at 40 °C to 15 min at 70 °C). Regarding its effect on polyphenols, infrared drying temperature showed a significant increase of total polyphenols’ content in dried leaves as compared to fresh ones whatever was the variety [29]. Other researchers [30] investigated the effect of a pilot-scale heat pump conveyor dryer using a response surface methodology to optimize the drying conditions. Optimum operating conditions were found to be at a temperature of 53.43 °C, air velocity of 0.64 m/s, and process time of 288.32 min. At this optimum point, total phenolic content loss, total antioxidant activity loss, final moisture content, and exergetic efficiency were found to be 9.77%, 44.25%, 6.0%, and 69.55%, respectively. The pilot-scale heat pump conveyor dryer has a low operating cost so it attracted the attention of investigators. Considering laboratory-type tray dryers, optimal values according to the response surface methodology for total phenolics, antioxidant activity, moisture content, and exergetic efficiency (10.25%, 41.88%, 6.0% and 65.50%, respectively) were obtained under a temperature of 51.16 °C with the air velocity of 1.01 m/s at 298.68 min.

Hot- and freeze- air drying by forced air laboratory dryer methods showed a significant effect on the concentration of the main polyphenols identified in leaves from the Serrana olive cultivar. Hot air drying provided a higher phenolic content, especially in oleuropein, than freeze drying. Drying at 120 °C was considered as the best processing condition [33].

Using a laboratory convective solar dryer, Bahloul *et al.* [26] found that the total phenols of olive leaves were significantly influenced by drying air conditions (temperatures: 40, 50, and 60 °C, and two drying air flow rates of 1.62 and 3.3 m^3^/min). In addition, the radical scavenging capacity was found to be higher in fresh leaves (with EC50 of about 40 μg/mL) than in dried ones (EC50 >50 μg/mL) [26]. In other research, microwave oven drying twice for 2 min at maximum power of 800 W and lyophilisation were adopted for oleuropein determination in olive leaves from Tunisian and French cultivars using mid-infrared spectroscopy [34]. The application of an ultrasound energy system was also investigated for drying olive leaves and it was reported that this could represent an interesting way to increase the drying rate [27].

## 3. Extraction Procedures

The extraction of bioactive compounds from plant materials is the first step in the utilization of phytochemicals in the preparation of dietary supplements or nutraceuticals, food ingredients, pharmaceuticals, and cosmetic products [35]. Thus, the development of “modern” sample-preparation techniques with significant advantages over conventional methods is likely to play an important role in the overall effort of ensuring and providing high-quality herbal products to consumers worldwide [36]. However, it is difficult to develop a single method for optimum extraction of all phenolic compounds due to the polarities of phenolic compounds varying significantly [37].

Studies on olive leaf reported the use of water, methanol, ethanol, acetone, as well as aqueous alcohol mixtures as the usual solvents for polyphenols’ extraction (Table 2).

**Table 2:** Extraction and analysis of phenolic compounds in olive leaves.

Extraction Technique	Analytical Technique	Observations	Reference
Extractant solvents: ethanol, methanol, acetone and their aqueous form (10%–90%, v/v). Extraction time: 24 h	HPLC-UV (280 nm) Stationary phase: C_18_ Lichrospher 100 analytical column (250 × 4 mm, 5 μm) at 30 °C Flow rate:1 mL/min Mobile phases: acetic acid/water (2.5:97.5) (A)and acetonitrile (B) Elution: gradient Total time: 60 min	70% ethanol as extractant solvent for high content of phenolics and antioxidant capacity. oleuropein (13.4%), rutin (0.18%). Silk fibroin was found to be a promising adsorbent for the purification of oleuropein and rutin from olive leaf extracts	[38]
30–50 mg of olive leaves powder	Mid-Infrared Spectroscopy Mid-infrared spectra were recorded between 4000 cm^−1^ and 700 cm^−1^ Nominal resolution was 4 cm^−1^	Mid-infrared spectroscopy, as a rapid tool, to predict oleuropein content in olive leaf from five Tunisian cultivars (Chemlali, Chetoui, Meski, Sayali and Zarrazi) Oleuropein: 8.72% and 17.95%	[34]
0.5 g of dry leaves extracted via Ultra-Turrax 10 mL of MeOH/H_2_O (80/20) Ultrasonic bath (10 min) Extraction repeated twice	HPLC-DAD-ESI-TOF- MS Stationary phase: Poroshell 120 EC-C18 analytical column (4.6 × 100 mm, 2.7 μm) (Agilent Technologies, CA, USA) Mobile phases: acidified in water (acetic acid 1%) (phase A) and acetonitrile (phase B) Elution: gradient Flow rate: 0.8 mL/min	30 phenolic compounds were identified. Total phenolic compounds: 52.12–60.64 mg/kg	[39]
Fresh leaves in aqueous methanol 80%	HPLC-DAD (240, 254, 280, 330 and 350 nm) MS (MSD API-electro spray) and NMR Stationary phase: Zorbax Stablebond SB-C18 column (5 µm; 250 × 4.6 mm (Agilent Technologies, Palo Alto, CA, USA) Mobile phases: acidified water (pH 3.2 by formic acid (A)), methanol (B) and acetonitrile (C) Elution: gradient Flow rate: 0.8 mL/min	Novel secoiridoid glucosides identified as a physiological response to nutrient stress	[40]
MAE 1.25 g of milled fresh olive leaves, 10 mL of methanol, ethanol and their aqueous form (40%–100%). Extraction time: 4–16 min, Irradiation temperature: 10–120 °C.	HPLC-ESI-TOF/IT-MS Stationary phase: C_18_ Eclipse Plus analytical column, Agilent Technologies, CA, USA) (4.6 × 150 mm, 1.8 μm) at 25 °C, Mobile phases: acetic acid (0.5%) (A) and acetonitrile (B) Elution: gradient Flow rate 0.8 mL/min	Univariate optimisation for phenolics extraction: methanol: water (80%) at 80 °C for 6 min 36 compounds	[41]
MAE Power 100–200 W, irradiation time 5–15 min, ethanol 80%–100%	HPLC-DAD (280, 330, 340 and 350 nm) Stationary phase: Lichrospher 100 RP_18_, Análisis Vínicos, Ciudad Real, Spain (250 × 4 mm,5 μm), Kromasil 5 C_18_ column, Scharlab, Barcelona, Spain (15 × 4.6 mm, 5 μm) Mobile phases: 6% acetic acid, 2 mM sodium acetate, in water (A) and acetonitrile (B) Elution: gradient Flow rate 0.8 mL/min GC-IT-MS ^#^ Stationary phase: fused-silica capillary column, Varian, TX, USA (VF-5 ms, 30 m × 0.25 mm, 0.25 μm)	Multivariate optimization for extraction of oleuropein and related biophenols: 200 W for 8 min, ethanol 80%, oleuropein 2.32%, verbacoside 631 mg/kg, apigenin-7-glucoside 1076 mg/kg, luteolin-7-glucoside 1016 mg/kg) Simple phenols were not found in the extracts obtained by MAE	[42]
USAE solvent concentration: 0–100% ethanol Ratio of solid to solvent: 25–50 mg/mL Extraction time: 20–60 min Frequency: 50 Hz	UV spectrometry (Folin–Ciocalteu)	Multivariate optimization: 50% EtOH, 500 mg dried leaf to 10 mL solvent, and 60 min Solvent concentration was proved to be the most significant parameter of all the parameters used	[43]
DUSAE (20 kHz, 450 W) Tested variables: probe position: 0–4 cm ultrasound radiation amplitude: 10%–50% Duty cycle: 30%–70% Irradiation time: 6–30 min Extractant flow- rate: 4–6 mL/min Ethanol: 50%–90% Water bath: temperature: 25–40 °C	HPLC–DAD (280, 330, 340 and 350 nm) Stationary phase: Lichrospher 100 RP_18_ (250 × 4 mm,5 μm), Kromasil 5 C_18_ column (15 × 4.6 mm, 5 μm) Mobile phases: 6% acetic acid, 2 mM sodium acetate, in water (A) and acetonitrile (B) Elution: gradient Flow rate: 0.8 mL/min GC-IT-MS Stationary phase: fused-silica capillary column (VF-5 ms, 30 m × 0.25 mm, 0.25 μm) Carrier gas: Helium (1 mL/min) Ionisation: electron impact	Multivariate methodology optimization: 1 g of milled leaves in a 59:41 ethanol–water mixture, bath temperature 40 °C, extraction time 25 min, ultrasonic irradiation (duty cycle 0.7 s, output amplitude 30% of the converter, applied power 450 W. Target analytes concentration:oleuropein, verbacoside, apigenin-7-glucoside and luteolin-7-glucoside contents: 22610 ± 632, 488 ± 21, 1072 ± 38 and 970 ± 43 mg/kg; respectively	[44]
SFE 1 g of milled olive leaves Pressure and temperature: 150 bar and 40 °C Extraction solvent: CO_2_ + 6.6% of ethanol as modifier Extraction time: 2 h	HPLC-ESI-TOF/IT-MS Stationary phase: C_18_ Eclipse Plus analytical column (4.6 × 150 mm, 1.8 μm) at 25 °C Mobile phases: acetic acid (0.5%) (A) and acetonitrile (B) Elution: gradient Flow rate 0.8 mL/min	Compared to other extraction techniques MAE, CM and PLE, SFE was the best extraction procedure for apigenin and diosmetin isolation	[45]
SFE Pressure: 30 MPa Extraction temperature: 50°C Separation temperature: 55°C Mode: dynamic Variables: solvent-to-feed ratio, 120 or 290; co-solvent: 5% or 20%	HPLC-DAD (248 nm) Stationary phase: SupelcoAnalytical Discovery HS C_18_ (250 × 4.6 mm, 5.0 μm) at 25 °C Mobile phases: H_2_O + 1% acetic acid (A) and MeOH (B) Elution: gradient Flow rate: 1 mL/min	Pressure: 30 MPa, extraction temperature: 50°C, separation temperature: 55 °C, mode: dynamic, solvent-to-feed ratio: 290, co-solvent: 20% Oleuropein 30%	[46]
PLE: 1 g of grinded olive leaves Solvent: ethanol or water Pressure 100 bar, temperature 150 °C time Extraction time: 20 min	HPLC-ESI-TOF/IT-MS Stationary phase: C_18_ Eclipse Plus analytical column (4.6 × 150 mm, 1.8 μm) at 25 °C Mobile phases: acetic acid (0.5%) (A) and acetonitrile (B) Elution: gradient Flow rate 0.8 mL/min MS: negative mode	PLE (using ethanol as solvent) produced the highest yield for all the studied varieties. PLE (using water as solvent) did not show a good efficiency either for extracting oleuropein.	[45]
PLE Ethanol (150 °C) Water (200 °C) Extraction time: 20 min	HPLC–ESI–QTOF–MS Stationary phase: C18 (3 μm, 2 × 150 mm) at 25 °C Elution: gradient elution program at a flow rate of 0.2 mL/min. The mobile phases consisted of water plus 0.5% acetic acid (A) and acetonitrile (B) MS: negative mode	The first time that lucidumoside C has been detected in olive leaves The ethanolic extract has proven to be especially rich in flavonoids, while the aqueous extract was richer in hydroxytyrosol.	[47]
PLE 6 g of grinded olive leaves Variables: temperature, static time, extraction cycles and EtOH (%) Pressure 1500 psi	HPLC-DAD (248 nm) Stationary phase: Supelco Analytical Discovery HS C_18_ (250 × 4.6 mm, 5.0 μm) at 25 °C Mobile phases: H_2_O + 1% acetic acid (A) and MeOH (B) Mode: gradient Flow rate: 1 mL/min	Multivariate optimization The extraction yield is mainly influenced by 3 factors (in the order of statistical significance): temperature, static time and extraction cycles. The effect is positive in all three cases. oleuropein: 26.1%	[48]
SHLE Tested variables: temperature, static and dynamic extraction time, extractant flow-rate and extractant composition	HPLC–DAD (280, 330, 340 and 350 nm) Stationary phase: Lichrospher 100 RP_18_ (250 × 4 mm,5 μm), Kromasil 5 C_18_ column (15 × 4.6 mm, 5 μm) Mobile phases: 6% acetic acid, 2 mM sodium acetate, in water (A) and acetonitrile (B) Elution: gradient Flow rate: 0.8 mL/min	Multivariate optimization 1 g of leaves , Pressure: 6 bar , 70:30 ethanol–water, temperature 140 °C, 6 min Dynamic mode,extractant for 7 min at 1 mL/min, Extraction time: 13 min 23 g/kg of oleuropein, 665 mg/kg of verbascoside, 1046 mg/kg of apigenin-7-glucoside, 998 mg/kg of luteolin-7-glucoside	[49]

^#^ Forthe characterization of simple phenols.

In recent works several techniques such as ultrasound-assisted UAE, supercritical fluid (SFE), pressurized liquid (PLE), and microwave-assisted (MAE) extraction have been used to isolate phenolic compounds from olive leaves (Table 2). Amongst the main advantages of such new techniques is the gain of extraction time, reduce of solvent volume, and enhance the extraction efficiency. Depending on the objective of the study, optimization of modern extraction techniques has been carried out. Univariate or multivariate optimization methodologies have carried out a detailed optimization of extraction of phenolic compounds from olive tree leaves.

Ultrasonic radiation is a powerful aid in accelerating various steps of the analytical process. Ultrasound can enhance the extraction processes and enable new commercial extraction opportunities and processes [37]. Cavitation favors penetration and transport at the interface between an aqueous or organic liquid phase subjected to ultra-sound energy and a solid matrix [44]. Dynamic ultrasound-assisted extraction multivariate optimization of variables such as probe position, ultrasound radiation amplitude, percent of ultrasound exposure duty cycle, irradiation time, extractant flow rate, extractant composition, and water bath temperature have been optimized for biophenol extraction from olive leaves [44].

Pressurized liquid extraction, or more commonly known by its trade name (accelerated solvent extraction), uses organic solvents at high pressures and temperatures above their normal boiling point to achieve fast and efficient extraction of the analytes from solid matrices. The nature of the solvent and composition, the solvent volume to sample mass ratio, extraction pressure and temperature, the number of extraction cycles, and the duration of each cycle are factors affecting the efficiency of the extraction. Most PLE applications reported in the literature employed the organic solvents ethanol and water [47,48,50].

Microwave-assisted extraction, also called the microwave-assisted process (MAP), has been applied in the development of extraction methods for phenolic compounds from olive leaves [41,42]. Among the conditions commonly studied for optimization of MAE process, the effects of solvent composition, solvent volume, extraction temperature, and matrix characteristics.

Super-critical fluid extraction has received increasing interest due to the reasons that super-critical fluids provide high solubility and improved mass-transfer rates and the operation being manipulated by changing the temperature or pressure.

Supercritical fluid extraction with CO_2_ is the most widely used solvent for SFE for its particular characteristics such as safety, non-toxicity, non-flammable, high selectivity, and moderate critical conditions (31.3 °C and 72.9 atm) [51]. However, this technique is limited to compounds of low or medium polarity [45]. In addition of modifiers, ethanol as a co-solvent is particularly useful to enhance the phenolic fraction yield [37]. The extraction pressure, the solvent strength of the fluid can be modified [51].

In a comparative study of different extraction techniques [45], each technique seemed to be more adequate than others for the extraction of each particular class of compounds. MAE and conventional extraction showed to be the choice for extracting more polar compounds, such as oleuropein derivatives, apigenin rutinoside, and luteolin glucoside. SFE-CO_2_ (using ethanol as a modifier) and PLE (using water as an extractant) did not show good efficiency for extracting oleuropein. However, SFE was the best extraction procedure for apigenin and diosmetin extraction.

## 4. Determination of Phenolic Compounds

The colorimetric Folin-Ciocalteu assay is the most used and rapid quantitative technique for the determination of total polar phenolics in olive leaves. For the determination of individual compounds, high performance liquid chromatography, mainly in the reversed-phase mode, coupled to several detectors (UV-VIS, MS, NMR, *etc.*) has been used being the UV-VIS the most used technique. Rare are the studies that reported the use of gas chromatography for the characterization of phenolics in olive leaves.

Columns most used in the HPLC analytical technique are C_18_ with 5 μm particle size. Shorter and narrower columns with small particle size are mostly preferred in order to obtain better resolution and reduce the time of analysis. Columns with 3 or 1.8 μm particle size were reported for separation of phenolics from olive leaves (Table 2). Gradient elution mode is commonly utilized. In fact, the complexity of the phenolic profile makes it not able to be well-separated by the isocratic elution mode.

Concerning the mobile phases, there is a wide range of possibilities; however, binary systems consisting of water and a less polar solvent, such as methanol or acetonitrile, are the most common mobile phases. Usually, acetic acid, formic or even perchloric acids are added to the aqueous phase to maintain a low pH and avoid phenolic dissociation [52].

UV-VIS detection systems continue to be one of the most used detection systems for phenolic compounds. The general use wavelength (280 nm) is preferred in most works. However, it should be considered that no universal absorbance maximum exists for olive leaf phenolics [15].

MS detection has also been employed for complete characterization of structurally-related compounds. Electrospray ionization in the negative mode was the most employed tool to determine phenolic compounds from olive leaves. MS provides higher selectivity than spectrophotometric detection. Furthermore, high-resolution mass analyzers offer the possibility to obtain structural information by accurate mass measurements by offering fragmentation patterns by MS/MS and MS^n^ experiments.

NMR was not commonly used for the determination of phenolic compounds in olive leaves. However it is a powerful technique for the structure elucidation of isolated compounds. Novel secoiridoid glucosides (6′-E-p-coumaroyl-secologanoside and 6′-O-[(2E)-2,6-dimethyl-8-hydroxy-2-octenoyloxy]-secologanoside were identified as a physiological response to nutrient stress in olive leaves suffering from boron deficiency [40].

## 5. Exploitation of Bioactive Components

Olive leaves, biomass produced in large quantities in the Mediterranean countries and particularly in Tunisia, should not be regarded as a bulky waste but as a resource that should be used. Much work has been done to try to use this byproduct and, thus, improve profitability in the olive sector.

An increasing interest has been given to natural antioxidants. Olive leaves a byproduct of high antioxidant potential has gained a big interest. Historically, olive leaves were totally oriented animal feed. However, they were also used in traditional herbal medicine for the treatment of certain diseases. Thanks to their richness in antioxidants, polyphenols, olive leaves were recently added to overripe olives at percentages of 2%–3% before the process to produce oils with more flavor and high oxidative stability [53]. On the other side, enrichment of oils with olive leaves, olive leaf extract, as well as the main secoiridoid compound oleuropein has been reported [25]. Supplementation of such extracts in the food industry may contribute to the health benefit of the consumers and also to enhance the stability of food products [54]. It was reported that the antioxidant capacity of olive leaf extract was higher than vitamin C and E or pure hydroxytyrosol, which is a strong antioxidant [9].

Moreover, extracts from olive leaves were recently marketed as dietary product (Briante *et al.*, 2002). These products are available as a complete dried leaves, powder, extract or capsules. During the recent years nutraceuticals are considered as health-promoting ingredients of food; thus, encapsulation can overcome challenges, such as degradation and oxidation reactions and provide them with necessary protection [55]. Encapsulation of olive leaf extract in β-cyclodextrin increased the aqueous solubility of the polyphenolic residue from olive leaf by more than 150% and can be used as a food additive [56].

## 6. Conclusions

Olive leaves are a byproduct of olive tree cultivation. Large amounts of leaves are collected during pruning, harvest, and processing. Available throughout the year, this biomass can be used as a cheap source of high added-value phenolic compounds. Phenolic composition of olive leaves is influenced by several factors which has been shown by the different treatment and analytical techniques used. Such bioactive ingredients could be used in medicines, pharmaceuticals, cosmetics, to improve the shelf life of foods, and to develop functional foods. Thus, valorization of olive leaves should be encouraged.
